# Building sustainable organizational capacity to deliver HIV programs in resource-constrained settings: stakeholder perspectives

**DOI:** 10.3402/gha.v6i0.22571

**Published:** 2013-12-13

**Authors:** Anjali Sharma, Philippe Chiliade, E. Michael Reyes, Kate K. Thomas, Stephen R. Collens, José Rafael Morales

**Affiliations:** 1 International Training and Education Center for Health (I-TECH), University of Washington, Seattle, WA, USA; 2 Liverpool Associates in Tropical Health (LATH), Liverpool School of Tropical Medicine (LSTM), Liverpool, UK; 3 Global HIV/AIDS Program, HIV/AIDS Bureau (HAB), Health Resource and Services Administration (HRSA), USA; 4 International Training and Education Center for Health (I-TECH), University of California San Francisco, San Francisco, CA, USA

**Keywords:** local partner, capacity building, participatory assessment, technical assistance, HIV/AIDS, program transition

## Abstract

**Background:**

In 2008, the US government mandated that HIV/AIDS care and treatment programs funded by the US President's Emergency Plan for AIDS Relief (PEPFAR) should shift from US-based international partners (IPs) to registered locally owned organizations (local partners, or LPs). The US Health Resources and Services Administration (HRSA) developed the Clinical Assessment for Systems Strengthening (ClASS) framework for technical assistance in resource-constrained settings. The ClASS framework involves all stakeholders in the identification of LPs’ strengths and needs for technical assistance.

**Objective:**

This article examines the role of ClASS in building capacity of LPs that can endure and adapt to changing financial and policy environments.

**Design:**

All stakeholders (*n*=68) in Kenya, Zambia, and Nigeria who had participated in the ClASS from LPs and IPs, the US Centers for Disease Control and Prevention (CDC), and, in Nigeria, HIV/AIDS treatment facilities (TFs) were interviewed individually or in groups (*n*=42) using an open-ended interview guide. Thematic analysis revealed stakeholder perspectives on ClASS-initiated changes and their sustainability.

**Results:**

Local organizations were motivated to make changes in internal operations with the ClASS approach, PEPFAR's competitive funding climate, organizational goals, and desired patient health outcomes. Local organizations drew on internal resources and, if needed, technical assistance from IPs. Reportedly, ClASS-initiated changes and remedial action plans made LPs more competitive for PEPFAR funding. LPs also attributed their successful funding applications to their preexisting systems and reputation. Bureaucracy, complex and competing tasks, and staff attrition impeded progress toward the desired changes. Although CDC continues to provide technical assistance through IPs, declining PEPFAR funds threaten the consolidation of gains, smooth program transition, and continuity of treatment services.

**Conclusions:**

The well-timed adaptation and implementation of ClASS successfully engaged stakeholders who committed their own resources toward strengthening organizational capacity. The sustainability of built capacity depends on continued investment in leadership, staff retention, and quality improvement.

Capacity-building efforts aim to realize the potential of nations and organizations to identify and solve problems in health systems through strong leadership and management, sufficient finances, and technical innovations (1–3). With shrinking funds at their disposal, donors have intensified their support to build local capacity to meet Millennium Development Goals (MDGs) related to maternal and child mortality, HIV, malaria, and tuberculosis more efficiently (4). In particular, they attempt to address weaknesses in health systems, human resources, and absorptive capacity that impede the achievement of the MDGs (2–4). Research shows that involving stakeholders to explicitly identify and address capacity gaps increases the likelihood that they can develop relevant, sustainable, country-owned health programs that lead to desired health outcomes (1, 3, 5–7).

This article examines the role of a participatory framework (where all stakeholders are involved at all stages, from the design of the assessment right through to the provision of technical assistance, where necessary) in building the capacity of organizations to deliver quality HIV care and treatment programs in resource-constrained settings. The impetus to develop the framework, which is called the Clinical Assessment for Systems Strengthening (ClASS; described further in this article), came from the US government's (USG) mandate for HIV care and treatment programs funded by the US President's Emergency Plan for AIDS Relief (PEPFAR (8)). The mandate required the transition of the management of these emergency programs from international organizations (referred to as international partners (IPs) in this article) to ‘local partners’ (LPs) capable of delivering a sustainable, country-owned program (i.e. a program with the capacity to maintain and adapt itself and its services, independent of major financial, managerial, and technical assistance from its original donor) (8–11). As defined by PEPFAR, LPs are legally registered governmental, non-governmental, academic, and privately owned organizations, and they are at least 75% owned, staffed, and managed by citizens or permanent residents of the host country (12, 13). Also, where Boards of Directors exist, the USG expects a membership composed of at least 51% citizens or permanent residents (12).

All the activities related to the ClASS framework from its early development to this assessment were funded under Cooperative Agreement U91HA06801 from the US Department of Health And Human Services, Health Resources and Services Administration (HRSA) to the International Training and Education Center for Health (I-TECH), University of Washington (UW). From 2008, HRSA worked with I-TECH to modify its participatory technical assistance framework, the Primary Care Assessment Tool (PCAT (14)). In 1998, HRSA developed the PCAT to allow on-site assessments of HIV primary care programs funded under the (then) Ryan White CARE Act. The tool included assessment of clinical, fiscal, administrative, and other health and support services modules, and it was successfully used to strengthen HIV primary care programs in the United States (14). HRSA and I-TECH modified the PCAT for use in international settings through an iterative process of stakeholder feedback, pilot testing in Uganda, and field testing in Nigeria (15). The ClASS framework, finalized in 2010, comprises five components: participatory approaches; expert-led review; modules for fiscal, administrative, and clinical assessment; targeted technical assistance; and guidance for implementing the ClASS framework (15).

From August 2010 to November 2011, key stakeholders implemented the ClASS framework to build the organizational capacity of 62 LPs in 10 countries through a comprehensive analysis of critical operational and technical areas. As per the ClASS framework, HRSA actively engaged with stakeholders (7), who defined the nature and scope of the review and participated in its implementation (15). Key stakeholders included in-country US Centers for Disease Control and Prevention (CDC) offices, IPs, country-specific LPs, and HRSA. Reviewers, expert in the subject area and trained in participatory approaches (of involving local stakeholders in all stages of the assessment), used ClASS modules to lead interviews, document reviews, and visit TFs supported by LPs. They purposively sought those likely to have the most information on the LPs’ managerial, financial, and technical capacity to deliver quality HIV care and treatment programs. They facilitated discussions on capacity strengths, capacity gaps, and action plans to address priority technical assistance needs. LPs decided on, and sourced, the technical assistance they needed to advance policy and program implementation, mechanisms for continuous learning, quality improvement, and partnerships (1). Stakeholders assessed progress through repeat ClASS visits as needed. In sum, the ClASS primarily helped develop capacity-building plans to guide future work and identify the technical assistance needed to strengthen the organizations’ capacity to provide quality HIV care and treatment programs that are sustainable (16).

Due to the short implementation period, the long-term impact of the ClASS framework on health systems and quality of services cannot yet be demonstrated (10). Still, ClASS-initiated changes in policy and practice, quality improvement, and consolidation of partnerships (1) should improve internal operations and lead to sustainable capacity building, where capacity-building activities are integrated into the program and are led, managed, and funded by local stakeholders independent of the original international donors (10, 11). Hence, this evaluation sought to understand what motivated organizations to implement ClASS-initiated changes and whether stakeholders believed that these changes were sustainable. Understanding the main elements and stakeholder perceptions of the sustainability of the capacity built through a participatory ClASS process could help donors to better support local ownership of priority health programs.

## Methods

### Materials, respondents, and data collection

From 28 November 2011 to 10 February 2012, the lead author conducted 42 group, individual, and phone interviews on ClASS outcomes with 68 stakeholders in Zambia, Kenya, and Nigeria (Table 1). One interviewee from Nigeria responded by e-mail due to scheduling constraints. All LP organizations in the three countries and TFs in Nigeria that had participated in ClASS processes were included. All stakeholders involved with ClASS planning, assessment, and implementation of ClASS-recommended changes were included. Nigeria had the first health program that had already transitioned to an LP. In Zambia, the LPs continued to receive technical support from IPs to manage HIV care and TFs. In Kenya, the LPs’ consortium members were not yet primary recipients of PEPFAR funds at the time of planning the evaluation.

**Table 1:** Number of organizations, interviews, and interviewees for ClASS by country

Country	No. of organizations	No. of interviews	No. of interviewees
Zambia	5	10	19
Kenya	6	8	24
Nigeria	10	25*	26*
**Totals**	**21**	**43**	**69**

* 1 by e-mail.

Interviewees were purposively sought (17) from among donor, IP, LP, and TF personnel to include leaders, managers, officers, and field staff who had some direct experience implementing the ClASS framework (18, 19). Organizations’ directors gave permission to interview the 58 identified staff. They also recommended interviews with 12 other staff members who, although not directly involved with ClASS planning and assessment, supported and implemented ClASS-recommended changes. Interviewees included 44 from nine LPs, 12 from four in-country IP offices, 7 from three in-country CDC offices, and, in Nigeria only, another 6 from four HIV/AIDS TFs. Due to travel restrictions, 25 Nigeria-based interviewees were interviewed by phone, and the remaining 44 interviewees were interviewed in person. While 32 interviewees agreed to individual interviews, the remaining 36 requested group interviews to reduce demands on their time. Only one person declined an interview due to time constraints.

Face-to-face interviews held at the interviewees’ convenience and with the aid of the referential interview guide lasted between 45 and 90 min. Phone interviews lasted between 20 and 45 min. The interview guide consisted of broad, open-ended questions to understand interviewees’ experiences (18, 20, 21) with the ClASS framework in order to improve ClASS-supported capacity building activities. Stakeholder accounts (18) were corroborated against ClASS repeat-visit assessment reports and triangulated with other in-country respondents’ accounts (19). The activities described in this article did not meet the US federal definition of human subjects research. As such, the University of Washington Human Subjects Division determined that human subjects ethics review and oversight were not required for these activities. Participants agreed to interviews for the purposes of improving the ClASS framework. Although information is provided by type of interviewee (i.e. LP, IP, TF, and the CDC), the views herein are not the official position of any of the organizations. No individuals are identified.

### Analysis

The interpretive phenomenological approach was used to collect and analyze data (18, 19, 21). The interpretive phenomenological approach is used to understand lived experiences and to articulate the nature, meaning, and impact of the experience on everyday actions (18, 21). First, individual experiences are studied, and then, through inductive reasoning, common meanings attached to those experiences are identified. All notes were read to identify core experiences and meanings and then re-read to compare interviewee experiences (18, 21). Recurring perceptions were compared to identify new and common themes with special attention being paid to strong examples of positive, neutral, and negative experiences with the ClASS implementations (18, 21). Themes were categorized using the theoretical lenses provided in select literature on sustainable capacity building and on scaling-up programs (2, 3, 5, 7, 10, 16, 22–29). Supporting quotes are interspersed throughout the text to illustrate stakeholder perspectives on the ClASS processes that worked and those that could be improved. These are labeled by type of interviewee (LP, IP, TF, CDC) followed by a serial number.

The qualitative methods described here are suitable for understanding phenomena in specific settings and are transferrable to other contexts. However, the findings are specific to individuals in these organizations and cannot be generalized to others who participated in the ClASS in other countries. Participants may have overstated positive and underplayed negative experiences with the ClASS framework. Non-verbal cues from interviewees in Nigeria were lost due to phone interviews and possibly reduced interviewee trust. The interviewer tried to assure trustworthy data by probing for descriptions and examples, triangulating information between interviewees and other sources of information, and paying close attention to non-verbal communication.

#### Main findings

LPs reported that implementing the ClASS framework led to changes in policy and practice, continuous quality improvement initiatives, and consolidation of partnerships, all of which improved internal operations (1, 10). From the LPs’ perspective, as voiced by LP1, ‘CIASS recommendations [had] become part of the basis for [organizational] capacity building’. LPs reported changes in governance, financial management, human resource, and grants management policies, procedures, and practices, among others (Table 2). TF3 reported similar improvements to internal operations as a result of ClASS visits:

We realized that though we are doing well, we could do better.… Now it's a lot better. Also recommendations in terms of quality … are in place.… That visit, it was very, very useful to us.

Table 3 summarizes the drivers, types, and conditions necessary to sustain strengthened capacities, which are further elaborated in this section.

**Table 2:** Types of actions taken by local partners across technical areas (only if identified as needed)

	Technical area								
	
	Administration				Finance			Clinical	
			
Types of actions taken	Gov	GM	HR	QI	BM	AS	CA	Sup	QoC
Updated policies, manuals and handbooks, and procedures to reflect standards and current practice	√	√	√	√	√	√	√	√	√
Instituted standardized processes and operating procedures	√	√	√	√	√	√	√	√	√
Systematic documentation of capacity-strengthening efforts	√	√	√	√	√	√	√	√	√
Elaboration on determining and declaring conflict of interest	√	√	√						
Improved performance evaluation tools and practices	√	√	√		√	√		√	√
Reassigned or contracted based on responsibility and productivity	√	√	√		√	√		√	
Improved monitoring and information systems	√	√		√	√	√	√	√	√
Improved reporting	√	√		√	√	√	√	√	√
Initiated new quality improvement cycles	√			√			√		√
Regularized verification of licences		√	√			√		√	
Revised sustainability and strategic plan	√				√			√	
Updated risk management and reduction plans	√				√			√	
Created structures for expedited decision making	√				√				
Improved procurement processes		√			√				
Realignment with the USG rules and regulations	√	√				√	√		
Improved referrals and linkages; community engagement									√
Systematic dissemination of best practices									√

Gov=governance; GM=grants management, HR=human resource, QI=quality improvement, BM=budget management, AS=accounting systems, CA=cost allocation, Sup=supervision, QoC=quality of care, USG=US government.

**Table 3:** Stakeholder perspectives on the drivers, types, and conditions necessary to sustain strengthened capacity

Drivers of change	Capacities strengthened	Conditions that sustain capacity
**Donor**	**Oversight**	**Internal environment**
● Interest	● Governance	● Organizational norms
● Funding opportunity	● Leadership	● Leadership commitment
● Commitment to local ownership	● Grants management	● Staff retention
**Local partners**	● Financial management	● Diversification of funding
● Desire for organizational growth and excellence	**Internal operations**	● Organizational influence
● Aspiration for improved patient outcomes	● Financial systems	● Advocacy
● Internal continuous quality improvement processes	● Management systems	● Continuous quality improvement
● Ownership of assessment	● Institutionalized policies	● Strategic partnerships
● Prioritization of capacity-building activities	● Standardized procedures	● Community involvement
● Resource mobilization	● Continuous quality Improvement	**External environment**
● Access to technical assistance	● Quality of care	● Political commitment
**International partners**	**Business development**	● Ministry of Health involvement
● Collaboration	● Strategic partnerships	● Supportive policy environment
● Technical expertise	● Applications aligned with donor expectations	● Stable economy
● Resource mobilization		● Investment in human resources for health
● Commitment to continuity of HIV programs		● Investment in infrastructure
**Participatory framework**		● Bridge funds to ensure consolidated gains
● Stakeholder engagement		
● Comprehensive review		
● Recognition of strengths		
● Professional discussions		
● Constructive solutions		
● Action planning		

### Donor interest and tangible funding opportunities

The first implementations of the ClASS framework coincided with the USG decision to issue competitive funding opportunity announcements to in-country programs. In this regard, a local partner commented that the implementation of the ClASS framework ‘was very timely’ (LP6). As an international partner (IP1) put it, the ‘ClASS brought out/reaffirmed commitment to transition.… The discussion/preamble helped everyone understand the HRSA/CDC expectations of local partners and of transition’.

All stakeholders noted HRSA's leadership in articulating the vision, engaging stakeholders, and committing time and resources (5, 7) for implementing ClASS as a technical assistance framework. An international partner (IP1) remarked that ‘the time and effort put in, shows the genuineness of HRSA’. A local partner (LP5) corroborated, ‘If the review is done by CDC/HRSA, it is taken very seriously’. Still, an international partner (IP3) cautioned against overcorrections:

When the donor asks … there is a tendency to fix things even if [they are] working for you. In that sense, ClASS can be a distraction. As it happens … [the ClASS] recommendations were useful.

### Comprehensive and compelling review

All interviewees acknowledged that the ClASS framework helped identify priority improvements that were needed in the critical areas of administration, financial management, and clinical services. The technical assistance needs that were identified were useful in planning and were ‘tailored to expectations’ for the effective management of the USG Cooperative Agreement (IP3). For this reason, LPs perceived the ClASS review as transformational, ‘an eye opener’ on the magnitude of ongoing HIV programs (LP1).

Stakeholders recognized that the ClASS framework had a ‘capacity building aspect that is … not common with many tools’ (LP7). The emphasis put on organizational strengths was ‘important as it [made] people more receptive and less defensive about areas that need to improve’ (LP1). Both IPs and LPs reported that ClASS reviewers stimulated LPs’ interest with novel but doable suggestions (5, 7). Reviewers convinced LPs through professional discussions that led to constructive solutions. As voiced by a local partner (LP2), ‘The approach is very friendly, fair and open-minded … the reviewers are very willing to listen to all arguments.… Top bottom approach is not helpful.…HRSA's approach is more sustainable as they continue in the learning process’.

### Ownership and collaboration

LPs felt that they *owned* the ClASS processes, being part of the discussions and planning on meeting recommendations arising from the ClASS review in their organization. One local partner (LP8) appreciated that in the ClASS framework, ‘Everything is done in a way that promotes ownership. You are the one who answers questions, shows documents; at the end, it is your plan too’.

LPs demonstrated motivation and leadership, and they acted on a genuine belief (5) that meeting capacity gaps would increase their ability to deliver quality HIV care and treatment (24–26). LPs were motivated by their desire to ‘excel’ and ‘to provide [the] best of service to patients and partners’ (LP7), while TFs were further spurred by the positive ‘feedback from patients themselves, [who] were appreciative as they experienced tremendous quality’ (TF1). LPs reported making most operational changes ‘in-house’, seeking ‘technical assistance only on an as-needed basis’ from IPs (LP6).

IPs served as larger anchor partners (5), having at least a year to promote, support, and share good practices among smaller partners. They provided (A) monetary support for staff recruitment and material procurements, (B) training on the use of new technologies or approaches, (C) manuals on policy and procedures for local adaption, and (D) technical review of LPs’ documents and implementation processes. IPs reported modifying their current processes and systems to facilitate progress and collaborating with LPs to share and create clinical assessment tools that identified and helped address frequent and common capacity gaps.

LPs and IPs also conducted and acted on their own, independent gap analyses to initiate improvements. As stated by an international partner member (IP2):

ClASS … helps, but it is not everything.

Local partners listed other partners and donors who provided technical assistance.

### Program and financial sustainability

The flexible yet standardized approach (23) to assess organizations’ fiscal, administrative, and program delivery systems (24), and to provide proactive technical assistance (25), helped stakeholders to meet the USG transition deadline of February 2012. In the context of transition, HRSA used the ClASS framework to strengthen LPs’ systems and their competitiveness to receive and manage PEPFAR funding. Stakeholders believed in the durability of the changes because they were ‘based on systems, not people’ (LP7) incorporated into the ‘organizational culture’ and response (LP8), and were ‘attitudinal, in processes and in knowing donor requirements’ (LP6).

Of the 62 LPs who participated in ClASS processes, 41 applied for and 39 (95%) received PEPFAR funding. While stakeholders could not rule out the role of the LPs’ preexisting accomplishments and reputation in their successful funding applications, complementary improvement activities supported by IPs and other donors also contributed to their successful applications for funding awards. One local partner (LP6) stated that:

We are known as serious, with proper skills and capacity. Sound in the financial, health, governance environment, we [were already] positioned to be able to [manage HIV programs]. ClASS and other donors look for the same things, only in different depth, so [ClASS efforts are] complementary.

LPs initiated some changes based on recommendations from the ClASS teams, for instance restructuring partner roles, which increased their ‘self-confidence’ to apply for PEPFAR funding. Stakeholders believed that following ClASS-based recommendations increased LPs’ competitiveness by increasing their ability to demonstrate strong systems to manage USG cooperative agreements. As stated by a CDC officer (CDC3), ‘If LPs did not know requirements, did not have systems, they wouldn't have been successful’.

One LP attributed their Global Fund sub-award to their human resources and administration systems, which had been strengthened as a result of the ClASS process and recommendations. Further, LPs, in some instances with active support from the CDC, were working to diversify their funding.

### Challenges to long-term sustainability

IPs and LPs gave voice to the vulnerability of HIV programs to continued staff attrition and weak political support. In spite of the participatory processes and the LPs’ determination, some recommendations did not apply, such as those on board registration in countries without such services, or changes in health care facilities under Ministry of Health management. Also, shortages in funding, personnel, or expertise constrained capacity-building actions. While the ClASS framework revealed strengths and shortcomings, ‘there was no transition budget’ (IP1) for making recommended changes. LPs with institutional affiliations faced bureaucratic processes and competing priorities, which required sensitization, advocacy, and creative problem solving by their leadership. Complex tasks also slowed progress. For example, successful transfer of data required prior placement of equipment, software, personnel, and security protections. Finally, competing tasks such as grant writing and implementing program activities delayed change.

The CDC had strategically funded IPs to continue providing technical assistance to LPs post transition. All stakeholders reported the need for the ClASS framework to use ‘scales/grading for what is acceptable given the context … to know readiness for transition’ (LP1) and ‘benchmarks … on which [IPs] base an exit strategy, else [LPs] are forever capacity building!’ (IP3). Competing priorities related to new funding negotiations, the closing and opening of grants, the need for additional clarification on role and function across partners, and instituting policies for cost allocation and expenditure had slowed capacity-building activities. Finally, local and international partners expressed concern about local partners’ ability to weather gaps in disbursement and funding in unstable financial and local environments, as voiced by LP1: You can have documents, constitution, manual and systems, but you need commitment to put things into practice … Organizations are vulnerable to gaps in funding where the program can deteriorate and keep on deteriorating … For instance, PMTCT, … when stocks are depleted, mums stop coming .… Sustainability is only possible through the government … They need funds from other partners.

For long-term sustainability, all interviewees stressed the need to include the Ministry of Health in ClASS to ensure that national interests were served, and to further funding opportunities through national systems.

## Discussion and conclusions

Implementation of the ClASS framework provided the momentum for change by addressing areas valued by LPs and facilitating innovation around building organizational capacity in HIV/AIDS programs (7). The ClASS visits created opportunities and fostered strategic partnerships that could strengthen health systems and program delivery (3). Shared data, experiential learning, and field visits allowed for joint decision-making and self-organizing processes (7, 9). LPs and IPs addressed policy systems and regulatory mechanisms simultaneously, and they continually linked them to organizational priorities to ensure relevance (6, 9). Crucially, LPs used their existing resources and organizational structures for building systems for continuous improvement (1). In-country IPs facilitated experiential learning (25) on implementing PEPFAR funded programs. The CDC ensured continuity in the transfer of knowledge, skills, and programs from IPs to LPs. Repeat visits allowed for ClASS processes to be informed by the results of the intervening actions (3). All of these aspects of the ClASS framework provided focus on the capacities to be built (9).

The ClASS framework met several criteria of sustainable organizational capacity building for scaling up programs, including having clear purpose and expected outcomes of the assessment and technical assessment processes; comprehensive assessment of operational and technical capacity, using participatory processes facilitated by an external reviewer; and technical assistance that builds on strengths and has the full commitment of and planned actions from all stakeholders (2, 3, 5, 7, 10, 18, 19, 26). In addition, in line with the Appreciative Inquiry method of organization development, the ClASS framework generated the positive affect, hope, aspirations, and authentic engagement that are required to increase stakeholder receptiveness to new ideas (5, 7). Reviewers being uninvolved in daily program activities (27) challenged stakeholders to reconsider current practices by revealing both their strengths and donor expectations (7). These processes motivated the organizations to make the normative and procedural changes (5, 7) needed to meet their organizational and programmatic goals (27).

The literature on sustainable capacity building (10, 28, 29) supports stakeholder contention that staff attrition and insufficient bridge funds threaten the consolidation of gains, smooth program transition, and continuity of services at TFs. To sustain changes in service delivery, ClASS teams should include the Ministry of Health and the end beneficiaries – the recipients of HIV care and treatment services (2, 28). Also, the stepwise process to systems strengthening warrants the development of benchmarks to monitor progress to the desired goal of sustainable, locally owned health programs (24). In the long term, the ClASS framework must be tested for health outcomes and predictability of success. Assessments of successes are complicated by short-term fidelity to ClASS recommendations and by changes in policies, external factors, personal engagement, and organizational norms in the long term (5, 6). Health outcomes, such as those related to morbidity, mortality, and quality of care, depend on both changes in health systems and necessary investments; for instance, changes in HIV/AIDS service-related supply chain management cannot be effective without investments in supplies such as antiretroviral therapy (6).

Courtesy bias, and for phone interviews loss of non-verbal information and reduced interviewee trust, remain possible limitations of the evaluation. Also, the choice of collecting views from three countries limits the full extent of the captured experiential learning to improve the ClASS framework. Still, interviewees’ views on select capacity-building processes and on the sustainability of the capacity built due to participatory action-oriented processes may be applicable to other programs being transitioned to country ownership in other settings. Positive and sustainable results depend on ongoing and sufficient investment in the infrastructure needed for quality improvement (5). LPs need implementation capacity, decision-making authority, and leadership to sustain meaningful change in their healthcare settings (5, 7).
